# Cell-Free Nucleic Acids in Cardiovascular Disease: From Biomarkers to Mechanistic Drivers and Therapeutic Opportunities

**DOI:** 10.3390/cells15010033

**Published:** 2025-12-23

**Authors:** Hannah Morgan, Keara Little, Suchandrima Dutta, Sophie Chen, Jiantao Gong, Siddu Koduri, Asma Raja, Wendy Lin, Kanishka Saini, Riya Bhullar, Wei Huang

**Affiliations:** 1 Division of Cardiovascular Health and Disease, Department of Internal Medicine, College of Medicine, University of Cincinnati, Cincinnati, OH 45267, USA; morganh6@mail.uc.edu (H.M.); littlek3@mail.uc.edu (K.L.); gongjt@ucmail.uc.edu (J.G.); kodurisa@mail.uc.edu (S.K.); rajaam@mail.uc.edu (A.R.); siyuan.lin@gmail.com (W.L.); sainika@mail.uc.edu (K.S.); bhullark@mail.uc.edu (R.B.); 2 Department of Pathology and Laboratory Medicine, College of Medicine, University of Cincinnati, Cincinnati, OH 45267, USA; duttasm@mail.uc.edu (S.D.); chen3sp@mail.uc.edu (S.C.)

**Keywords:** cardiovascular disease (CVD), heart failure (HF), cell-free nucleic acids (cfNAs)

## Abstract

**Highlights:**

**What are the main findings?**

- Cell-free nucleic acids (cfNAs)—including cfDNA and cfRNA—are continuously released from both healthy and diseased cells into the bloodstream and other biofluids, where they circulate as short fragments bound to proteins or packaged within extracellular vesicles.
- cfNAs can be collected noninvasively (e.g., blood, urine) and are consistently elevated in heart failure (HF) and across a wide range of HF-associated cardiovascular diseases (CVDs).

**What are the implications of the main findings?**

- cfNAs provide sensitive, early, and mechanistic indicators of disease onset and progression. Integrating cfNA profiling with established biomarkers and imaging modalities enhances diagnostic accuracy and enables more precise, individualized patient management.
- Noninvasive detection and therapeutic targeting of cfNAs represent a shift in clinical practice from symptom-based treatment toward mechanism-directed intervention, enabling direct modulation of the molecular drivers of HF and broader cardiovascular pathology.

**Abstract:**

Cardiovascular disease (CVD) remains the leading cause of morbidity and mortality worldwide, with heart failure (HF) representing a major contributor to hospitalizations, healthcare costs, and death. Effective management of HF is hindered by the limitations of current biomarkers and diagnostic tools. Conventional biomarkers, such as natriuretic peptides, primarily reflect downstream hemodynamic stress and often lack specificity, particularly in HF with preserved ejection fraction or multiple comorbidities. While imaging provides valuable structural and functional information, it is resource-intensive, costly, and unsuitable for frequent longitudinal monitoring. As a result, these conventional approaches are inadequate to capture the dynamic and heterogeneous nature of HF pathophysiology. Circulating cell-free nucleic acids (cfNAs), including cell-free DNA (cfDNA) and RNA (cfRNA), have emerged as promising noninvasive liquid biopsy biomarkers capable of providing real-time insight into upstream pathological events, such as cardiomyocyte injury, immune activation, inflammation, and maladaptive remodeling. Importantly, cfNAs also act as active mediators of CVD pathology. When released under stress or injury, cfNAs interact with pattern recognition receptors (PRRs) that trigger sterile inflammation, cardiovascular cell dysfunction, and adverse cardiac remodeling. This review summarizes the origins, mechanistic roles, and clinical significance of cfNAs in HF and related CVD, highlighting their dual roles as diagnostic biomarkers and mechanistic effectors of disease. Finally, we discuss emerging cfNA-targeted therapeutic strategies, challenges, and future opportunities for precision medicine in HF and HF-associated CVD.

## 1. Introduction

CVD represents the most significant global health burden, accounting for nearly one-third of deaths each year and remaining a leading cause of morbidity worldwide. Although substantial progress has been made in prevention, diagnostics, and therapies, the overall burden of CVD continues to rise, largely driven by population aging, lifestyle factors, and associated comorbidities [1,2]. Among these conditions, heart failure (HF) represents a major public health challenge, affecting over 55 million people worldwide, and contributes substantially to morbidity, mortality, and healthcare expenditures [3]. Despite advances in pharmacological and device-based therapy, prognosis remains poor; approximately 30% of patients die within the first year of diagnosis, with mortality risk further escalating with advancing age [4,5]. HF is a complex clinical syndrome characterized by the heart’s inability to pump sufficient blood to meet the metabolic demands of the body [6,7], and it arises from diverse etiologies, including ischemic events, hypertension, cardiomyopathy, and other chronic conditions [8]. This etiological and pathophysiological heterogeneity of HF underscores the inadequacy of current diagnostic and prognostic approaches, which heavily rely on clinical parameters and conventional biomarkers [9,10].

Among these biomarkers, natriuretic peptides (BNP and NT-proBNP) are widely used in HF diagnosis and monitoring [11]. While they provide valuable information about hemodynamic stress, their diagnostic specificity is compromised, as their levels are significantly influenced by comorbidities, including renal dysfunction, obesity, and pulmonary disease [11,12,13]. In some HF patients, particularly those with preserved ejection fraction, natriuretic peptide levels remain within the normal range, further limiting their utility for early diagnosis or risk stratification [12]. These biomarkers largely reflect downstream hemodynamic stress rather than upstream molecular mechanisms, such as cardiomyocyte injury, immune activation, and inflammation, that drive HF onset and progression [10,14]. While imaging procedures can provide complementary structural and functional insight, they are costly, resource-intensive, and impractical for serial monitoring. Critical mechanistic insights of HF pathology have often required invasive biopsies or postmortem tissue analysis, which are impractical for longitudinal disease tracking [15]. These limitations highlight the urgent need for novel biomarkers that are noninvasive, real-time, mechanistically informative, and feasible for repeated monitoring.

Recent research highlights cell-free nucleic acids (cfNAs), including circulating cell-free DNAs (cfDNAs) and cell-free RNAs (cfRNAs) obtained from plasma and other biofluids via liquid biopsies, as promising next-generation biomarkers. The concept of cfNAs first originated in cancer, when Mandel and Métais discovered cfNAs in the plasma of cancer patients in 1948 [16]. However, due to molecular technique limitations, they were overlooked, until 1997, when male fetal-derived DNA was found in maternal plasma/serum by PCR, which established the feasibility of cfNA for noninvasive clinical application [17]. These findings laid the foundation for modern noninvasive prenatal testing (NIPT) (sex determination, RHD genotyping, and later aneuploidy screening via sequencing) [18]. With the development of high-throughput sequencing technologies, the clinical application of cfNAs has been rapidly growing, particularly in the field of oncology for diagnosis, molecular profiling, assessing drug resistance, and the monitoring of therapies in patients with metastasis [19,20,21,22]. Building on these successes, cfNAs are now increasingly studied in other diseases, including cardiovascular conditions. For example, in heart transplantation, donor-derived cfNAs have been used for noninvasive rejection surveillance, which reduces routine biopsy frequency [23,24].

By integrating mechanistic specificity with clinical accessibility, cfNAs have unique advantages as biomarkers because they are obtained through noninvasive liquid biopsies, feasible for serial monitoring, and capable of providing both diagnostic and prognostic information. More importantly, cfNAs are not only the passive biomarkers; they also provide molecular-level insight about key processes such as cardiomyocyte injury, inflammation, and remodeling [25,26], which are central to HF progression, offering a powerful framework for advancing precision HF management. This review synthesizes current knowledge on the biological origins, mechanistic contributions, and clinical relevance of cfNAs in various HF-associated diseases, while also exploring their therapeutic potential.

## 2. Definition of cfNAs (cfDNAs and cfRNAs)

**Figure 1:**
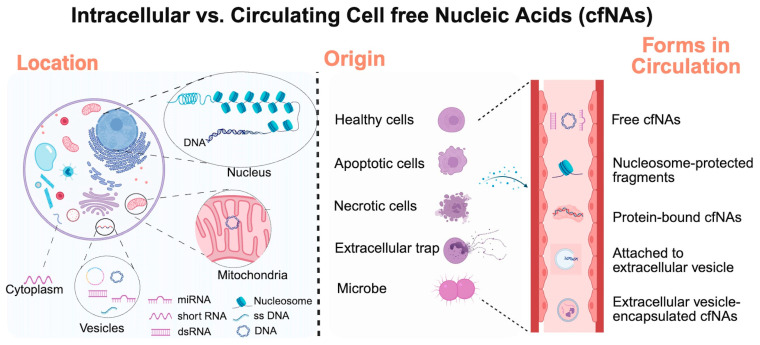
Overview of various intracellular and cell-free nucleic acids (cfNAs). Created by Biorender.

CfNAs originate from both healthy and diseased cells and are released into circulation and other biofluids [22]. Unlike intracellular NAs, cfNAs are present in the extracellular compartment as short fragments that are either (1) bound to proteins (e.g., nucleosomes for DNA; ribonucleoproteins for RNA) or (2) encapsulated in extracellular vesicles (EVs), such as exosomes, microvesicles, or apoptotic bodies [27,28]. These structural contexts protect cfNAs from degradation and make them morphologically and biophysically distinct from cellular NAs (Figure 1).

Elevated levels of circulating cfNAs have been increasingly observed under pathological conditions and clinical diseases. CfNAs can be categorized by both their origins (nuclear vs. mitochondrial) and their types (DNA vs. RNA) (Table 1, Table 2, Table 3 and Table 4). Among these, nuclear cfDNAs (cf-nDNA) generally reflect cell death and tissue injury, whereas mitochondrial cfDNA (cf-mtDNA) acts as a potent damage-associated molecular pattern (DAMP) due to its bacterial ancestry and unmethylated CpG motifs, linking it to a strong activator of innate immune pathways [29]. Similarly, cfRNAs, including messenger RNA (mRNA), microRNA (miRNA), long noncoding RNA (lncRNA), and circular RNA (cirRNA) can act as markers of cellular stress and inflammation, as they are increasingly recognized as mediators of intercellular communication and immune activation [28,30]. Together, cfDNAs and cfRNAs represent both diagnostic indicators and functional effectors of CVD processes.

## 3. cfNAs as Molecular Signatures of Cardiac Cell Injury and Death

Cardiomyocyte loss is a key driver of CVD pathogenesis and disease progression due to the limited regenerative capacity of cardiomyocytes. Excessive cardiomyocyte death leads to various CVDs, including myocardial infarction, arrhythmia, HF, and sudden cardiac death [56], which is strongly associated with CVD morbidity and mortality. Therefore, accurate detection and monitoring of cardiomyocyte death is crucial for the diagnosis and management of heart disease, particularly in patients with acute myocardial infarction (AMI). However, current noninvasive approaches remain limited in their ability to capture the dynamics of cardiomyocyte death in vivo. Although cardiac troponins (cTn) are widely accepted clinical markers of cardiac injury, they have key limitations. (1) They cannot specify the etiology (ischemia vs. non-ischemia) and must be interpreted with the clinical context carefully [57]. (2) cTn levels may rise in healthy individuals after intensive exercise or in chronic kidney disease, where impaired kidney clearance and ongoing myocardial stress confound the diagnosis [58,59]. (3) cTn levels do not quantify the extent of cardiomyocyte death and may rise in response to reversible injury [60]. Although high-sensitivity cTn assays have improved early detection (within 3 h) [61,62], the cTnT levels remain elevated for up to 14 days after MI, limiting their application for monitoring ongoing injury or treatment response [63].

In contrast, circulating cfDNA acts as a rapid and mechanistically direct indicator of cell death. In healthy conditions, most of the cf-NAs in blood originate from hematopoietic cells [64]. Under disease, tissue-derived cfDNA accumulates in the circulation, which results from increased cell death within the injured organ. During apoptosis or necrosis, nuclear chromatin [65] is cleaved into characteristic nucleosome-protected DNA fragments. These fragments are subsequently released into the circulation and trimmed by circulating extracellular nucleases, generating stable cfDNAs detectable in plasma, urine, and other biofluids [66]. Clinical studies demonstrate that cfDNA rises rapidly following cardiomyocyte death. In patients with myocardial infarction, cfDNA increases within 0–2 h after the onset of chest pain, with concentration increasing up to 50-fold [67,68]. Peak cfDNA levels strongly correlated with peak troponin, supporting cfDNA release as a direct consequence of acute cardiomyocyte death. In patients with ST-elevation myocardial infarction, cfDNA levels also correlate with infarct size and worse prognosis [69]. Importantly, unlike cTn, cfDNA levels are not elevated in patients with kidney dysfunction [70,71], as it is primarily cleared by the liver and spleen [27], positioning cfDNA as a more specific and reliable marker in individuals with impaired renal function.

Moreover, circulating nucleosome-protected cfDNAs retain cell type-specific epigenetic features, such as nucleosome footprints and cytosine (CpG) methylation patterns [72]. Beyond quantitative changes, comparing cfDNA methylomes to reference tissue atlases enables precise tissue-of-origin mapping [72,73,74,75], which further broadens its diagnostic application in CVDs. Comparative methylome analysis in 23 human cell types/tissues identified unmethylated FAM101A as a specific cardiomyocyte cfDNA marker, allowing differentiation between myocardial injury and non-cardiac injury [69]. In patients with ST-elevation myocardial infarction, which is characterized by acute cardiomyocyte death, robust cardiac cfDNA signals have been detected. Elevated cfDNA level correlates strongly with established biomarkers such as cTn and creatine phosphokinase (CPK) [69]. In pediatric congenital heart disease, FAM101A cfDNA has been shown to detect cardiomyocyte injury earlier than cTn [76]. Although the sample size is limited, it highlights the clinical promise of cfDNA to trace cardiomyocyte injury. Emerging methods such as cfDNA nucleosome chromatin immunoprecipitation and sequencing (ChIP–seq) [77] have been developed to detect histone modification signatures retained on cfDNA fragments, offering additional resolution for cell-of-origin identification. Future work is needed to characterize methylation signatures across cardiac subtypes and disease states to further refine cfDNA-based diagnostics.

Mitochondrial cfDNA (cf-mtDNA), as another major source of cfDNA, has been recognized as an additional marker of cell injury and death. Due to the lack of nucleosome-associated histone proteins in mitochondria, cf-mtDNA release is faster and more abundant compared to cf-nDNA [78,79], which indicates that cf-mtDNA might serve as a more sensitive diagnostic tool. In a study led by Konstantin A. Krychtiuk and colleagues [41], plasma cf-mtDNA levels were measured in 90 patients with severe acute HF. The cf-mtDNA levels were elevated sharply, and the patients in the highest quartile had a 3.4-fold increased risk of short-term mortality, independent of other risk factors (renal function, NT-proBNP). Although the study did not specify the cell/tissue resource of these elevated cf-mtDNA, the rapid elevation of cf-mtDNA suggests cf-mtDNA might be a biomarker for acute cardiomyocyte damage or death, given the high mitochondria enrichment in cardiomyocytes. Interestingly, in tachycardia-induced cardiomyopathy (TIC), cf-mtDNA levels were markedly reduced compared to those without TIC, while nuclear cfDNA levels remained unchanged [80]. These findings suggest cf-mtDNA may function as a stage-dependent, bidirectional biomarker in CVD, from mitochondrial depletion in chronic remodeling to excessive release during acute decompensation. Unlike cf-nDNA, cf-mtDNA generally exhibits greater size variability [39,40], it is necessary to establish standardized blood processing and quantification protocols. Despite growing evidence that cf-mtDNA reflects mitochondrial stress and predicts cardiovascular outcomes, its clinical utility remains limited by methodological heterogeneity, lack of tissue/cell specificity, and uncertain mechanistic causality. Integration of cf-mtDNA with intracellular mt-DNA copy number [81] and functional assays, and longitudinal, multi-center studies, is essential to define cf-mtDNA as a robust biomarker in HF and associated conditions.

The cardiovascular system contains diverse non-cardiomyocyte populations, including endothelial cells. Endothelial cell injury contributes to vascular dysfunction and CVD-associated high-risk conditions, such as chronic kidney disease [82], hypertension [83,84], and atherosclerosis [85]. In healthy individuals, hematopoietic cells and vascular endothelial cells are major sources of circulating cfDNA [64]. Growing evidence suggests that cfDNA signatures can reflect endothelial injury across vascular pathologies. Amanda et al. reported that cfDNA levels correlated with established markers of endothelial dysfunction in patients with chronic kidney disease, a major risk factor of CVD, and this elevation is independent of renal impairment and systemic inflammation [71]. Similarly, in hemodialysis patients with or without diabetes mellitus, cfDNA concentration was found to be strongly associated with vascular injury, especially in diabetic patients, rather than proinflammatory conditions [86]. In another study with pulmonary arterial hypertension (PAH) patients, a higher level of cfDNA was observed compared with the healthy controls. Cell-of-origin assay of cfDNA revealed a significant contribution of vascular endothelial cells in PAH patients [87]. In atherosclerosis (AS) [88], cfDNA concentrations correlate with plaque development and disease progression. These findings demonstrate that cfDNA acts as a broad indicator of vascular and cardiac cell injury, not limited to cardiomyocyte injury and death.

Along with the increasing application of cfDNAs, increased attention has been directed toward cfRNAs in the context of cardiovascular diseases [89]. Unlike DNA, cfRNAs lack nucleosome shield protection and are more susceptible to degradation by circulating RNase. As a result, their concentration in blood is significantly lower and more variable, often ranging from pg/mL to ng/mL [90]. To maintain stability, most cfRNAs are not freely circulating but are packaged within extracellular vesicles, bound to RNA-binding proteins, or assembled with lipoproteins [89]. These protective mechanisms not only protect cfRNAs from rapid enzymatic degradation but also facilitate intercellular transport and uptake. In the clinic, cfRNAs have demonstrated promising diagnostic and mechanistic potential. As the most abundant cfRNA, cf-ribosomal RNA (cf-rRNA) has been shown to increase drastically in ischemia–reperfusion injury and originates predominantly from damaged cardiomyocytes [91]. These released cf-rRNAs promote inflammation [91], vascular permeability [92], and blood coagulation [93], suggesting their roles in CVD pathogenesis and their utility as a biomarker of acute cardiomyocyte injury. Among cfRNAs, cf-miRNAs are the most extensively studied. Although it has been well established that miRNAs are critical regulators of cardiovascular biology, their clinical application as biomarkers for CVD is inconsistent due to low abundance, variable stability, and methodological heterogeneity [94]. Multiple studies have investigated the diagnostic utility of miRNA in CVD, including myocardial infarction [95,96,97] and HF [98,99,100,101,102]. Wong et al. initiated a large multicenter study involving 1710 participants, including a discovery cohort (180 HFrEF, 158 HFpEF, and 208 non-HF cases), and two sizable independent cohorts from Singapore (241 control subjects and 207 HF; HF subtype categorization: 116 HFrEF and 72 HFpEF) and New Zealand (358 control subjects and 358 HF; HF subtype categorization: 145 HFrEF and 179 HFpEF) [103]. They demonstrated that the plasma multi-miRNA panel, particularly when combined with NT-proBNP, accurately detected non-acute HF and differentiated HF subtypes (HFpEF vs. HFrEF). These findings underscore strong clinical relevance and the value of integrating cfRNA with established biomarkers to improve diagnostic precision. Despite this promise, cfRNA quantification and characterization are challenging due to the low abundance and poor stability [94]. Recent studies have increasingly explored extracellular vesicle-derived miRNAs, which display enhanced stability and cell type specificity [104]. The cell-specific EVs could serve as predictive biomarkers or enable longitudinal monitoring of patients across different stages of CVDs. Future research should integrate deep cfRNA sequencing with tissue-resolved transcriptomics to trace the cellular RNA origins and dynamics of cfRNA, as well as establish standardized pre-analytical and analytical protocols.

Together, these cfNA species provide complementary molecular perspectives on cardiac injury. Analysis of cfNA profiles not only enables the detection of injury but also reveals its etiology, timing, and cellular source, thereby advancing mechanism-informed and dynamic assessment of cardiac damage.

## 4. cfNAs and Cardio-Immunology

Cardio-immunology has rapidly expanded in recent years, driven by growing recognition of the immune system’s role in CVDs. The first concept was shaped in the 1990s when early studies revealed that patients with HF exhibited elevated circulating levels of proinflammatory cytokines such as tumor necrosis factor (TNF), interleukin-1β (IL-1β), and interleukin-6 (IL-6), compared with healthy individuals [105,106,107]. However, these proinflammatory cytokine increases were nonspecific, reflecting a common response to all forms of myocardial injury, which could not specify the etiology of cardiac injury. Thus, the observation of cardio-immunology was initially underappreciated. Later, the field accelerated with the discovery of innate immunity, where the pattern recognition receptors (PRRs) can sense the damage-associated molecular patterns or pathogen-associated molecular patterns (DAMPs/PAMPs) and trigger downstream inflammatory cascades [108,109,110]. Among the most potent DAMPs are NAs released from the stressed or dying cells. Circulating cfNAs can engage PRRs such as Toll-like receptors and cytosolic sensors, triggering sterile inflammation. The immunogenic activity of cfNAs provides a potential mechanistic framework for understanding how inflammation is activated in cardiovascular disease, thereby providing a mechanistic bridge from myocardial injury to immune activation and adverse cardiac remodeling in HF progression.

### 4.1. cfNAs in Extracellular Traps

During inflammation, sustained activation of innate immune cells can result in the release of extracellular traps (ETs) [111]. The web-like ETs contain chromatin, histones, and granule proteins that capture, neutralize, and eliminate pathogens. While neutrophils are the primary cells responsible for ET release, macrophages and dendritic cells can also generate these structures [111,112]. Because ETs contain abundant nuclear NAs, mitochondrial NAs, and small RNAs, they present a major potential source of circulating cfNAs. Moreover, the proteins contained in the ETs protect these NAs from nucleases degradation and prolong their bioavailability in circulation [113,114]. Under pathological conditions, when ET formation becomes excessive or dysregulated, ET-derived cfNAs act as DAMPs that amplify inflammation and contribute to tissue injury.

Neutrophil extracellular traps (NETs), the most studied form of ETs, are formed through a process called NETosis, which occurs via two canonical programs, lytic (suicidal) and non-lytic (vital) NETosis. Both forms of NETosis release substantial amounts of extracellular cfNAs, including cfDNA [115,116,117] and cf-mtDNA [118], which have been implicated in systemic immune dysregulation in disease [119]. Moreover, cfDNA levels strongly correlate with NET formation across diverse diseases [9,120], indicating NETs as a key contributor to the cfDNA pool. NET-derived cfDNA may also be involved in intercellular communication through vesicle-mediated pathways, as cfDNA has been detected within exosomes in human plasma samples [121]. Despite the central role of NETs, they are not the sole source of the circulating cfNA pool. Cardiomyocytes, endothelial cells, and fibroblasts under stress or injury also release cfNAs through apoptosis, necrosis, or active secretion. Thus, while NETs provide a major spike in cfNA release during acute inflammation, cfNAs from various cell types collectively serve as key messengers linking cellular injury to innate immune activation. This concept provides a foundation for understanding how cfNAs interact with PRRs and initiate downstream inflammatory cascades in CVD.

### 4.2. Interaction of cfNAs with PRRs and Application

Emerging evidence shows that PRRs are the primary innate immune sensors that detect cfNAs and initiate inflammatory responses, including type I interferon production, NF-κB activation, and inflammasome responses. PRRs are categorized into two major classes based on their subcellular localization, enabling the immune system to detect ‘outside-in’ versus ‘inside-out’ danger signals and activate distinct inflammatory cascades. The membrane/endosomal PRRs (e.g., TLRs and C-type lectin receptors) recognize ‘outside-in’ cfNAs via endocytosis and activate MyD88/TRIF-dependent pathways, leading to downstream NF-κB/IRF3/7 activation, with specific ligand recognition such as TLR3 for dsRNA, TLR7/8 for ssRNA, and TLR9 for CpG DNA) [122,123]. In contrast, the cytosol PRRs (RIG-I-like receptors, RLRs, NOD-like receptors (NLRs), and absent-in-melanoma 2, AIM2) monitor the ‘inside-out’ cfNAs from organelle leak or endosomal escape [124]. RLRs primarily activate interferon pathways, while NLRs and AIM2 assemble inflammasome complexes that drive caspase-1 activation and proinflammatory cytokine release [122,125,126,127,128,129].

Multiple studies have demonstrated a strong association between NET, and PRR activation in diverse disease states such as COVID-19, acute kidney injury, lupus, cancer, ST-elevated myocardial infarctions, and psoriasis [130,131,132,133,134]. Importantly, within the cardiovascular system, NET-derived cfNAs and associated proteins function as potent PRR ligands [135]. cfDNA engages TLR9 on cardiomyocytes and macrophages, driving proinflammatory signaling and adverse remodeling [136]. NET-associated histones activate TLR2 and TLR4 on endothelial cells, leading to endothelial injury, barrier dysfunction, and vascular inflammation [137]. NET-derived mtDNA is recognized by cGAS–STING and AIM2 inflammasomes in cardiac immune cells, triggering type I interferon and IL-1β production [138]. In addition to NET cfDNA, NET-associated RNAs have also been shown to activate specific PRRs (TLR7 and TLR8), amplifying innate immune response and inflammatory cascades [139]. NET-associated extracellular RNAs were demonstrated to act as a composite DAMP that promotes inflammation by further amplifying neutrophil activation and the release of more NETs [134]. Collectively, these findings indicate that NET-derived cfNAs as not only biomarkers of cardiovascular inflammation but also active mediators of PRR-driven signaling that exacerbate cellular dysfunction, promote adverse remodeling, and accelerate CVD progression.

## 5. Clinical Significance of cfNAs Across HF-Related Conditions

Circulating cfNAs reflect pathological processes and offer a valuable approach for distinguishing diverse etiologies of HF. In this section, we discuss the clinical relevance of cfNAs across the major cardiac conditions that contribute to HF development. These conditions were selected for their prevalence and mechanistic diversity, which span ischemic, metabolic, structural, inflammatory, and electrical dysfunctions in the heart. By analyzing cfNA profiles within these contexts and integrating the findings with established diagnostic modalities, cfNAs have promising potential to enhance disease classification, improve diagnostic precision, and guide therapeutic strategies, ultimately advancing clinical management in HF-associated diseases.

### 5.1. Ischemic Injury (Myocardial Infarction)

Myocardial infarction (MI) triggers HF through irreversible cardiomyocyte loss, leading to adverse ventricular remodeling characterized by inflammation, fibrosis, and contractile dysfunction [68]. Ischemic injury causes cardiomyocyte necrosis, resulting in the release of cfNAs [140]. The early and accurate diagnosis of AMI is crucial for timely treatment and improved patient outcomes. To determine the potential role of cfDNA as an early biomarker in AMI patients, Tan et al. initiated a systematic review and meta-analysis involving 1804 participants (819 AMI and 985 control subjects without AMI) across five countries. Despite methodological heterogeneity in AMI-defining criteria and cfDNA quantification, six studies (496 AMI, 308 control) consistently showed markedly elevated circulating cfDNA in AMI patients (standardized mean difference ~3.47), with strong diagnostic performance (sensitivity ~87%, specificity ~96%, AUC ~0.96). Importantly, peak cfDNA levels, the highest concentrations measured during the clinical course, correlated with peak cTn in two studies, supporting cfDNA release as a direct consequence of acute cardiomyocyte necrosis. In the clinic, patients with kidney dysfunction have significantly higher mortality following AMI [141]. However, traditional cTnT cut-off values have limited specificity for diagnosing AMI due to elevated cTn levels in chronic kidney disease [142]. In contrast, cfDNA may provide improved diagnostic accuracy in this population due to its early release and clearance by the liver and spleen [27]. Circulating miRNAs have also shown strong potential as early AMI biomarkers that are also upregulated post MI and can be detected in the circulation, where their levels correlate with infarct size, left ventricular ejection fraction, and long-term prognosis [140]. Several miRNAs (hsa-miR-1-3p, hsa-miR-19b-3p, hsa-miR-208a, hsa-miR-223-3p, hsa-miR-483-5p, and hsa-miR-499a-5p) peak within 4 h after MI and exhibited better diagnostic sensitivity compared to cardiac cTns and creatine kinase-MB [143]. Notably, some miRNAs (hsa-miR-21, hsa-miR-133, and hsa-miR-499) help distinguish acute coronary syndromes from stable CVD [144], with miR-499 highly correlating with myocardial damage severity. In addition, cfRNAs such as miR-21 and miR-155 modulate fibroblast activation and macrophage polarization, thereby influencing immune and fibrotic pathways during post-MI repair [140]. Together, early peak expression, high sensitivity, and strong specificity position cfNAs as potential biomarkers for AMI. However, current studies are limited by small sample sizes, sample selection bias (excluding patients with comorbidities), variable sampling times, and heterogeneity in cfNA quantification methods. The temporal relationship between cfNA release, infarct size, and remodeling remains incompletely defined. Future studies should employ standardized longitudinal sampling and multi-omic integration to validate cfNA dynamics for early MI diagnosis and post-infarction prognosis. The recent concept of ‘peak cfRNA’ in cancer studies [44] presents a novel method combining statistics and machine learning models to identify tumor-derived transcripts, which offers similar transformative potential for myocardial infarction and CVD.

### 5.2. Metabolic Disease (Diabetic Cardiomyopathy)

Diabetic cardiomyopathy, a major complication in type 2 diabetes mellitus (T2DM), develops independently of coronary artery disease, driven by hyperglycemia, insulin resistance, and lipotoxicity. These metabolic disturbances induce oxidative stress, low-grade inflammation, and myocardial fibrosis [145]. Elevated oxidative stress contributes to increased levels of both cf-mtDNA and cf-nDNA [47], which further activate inflammatory pathways and exacerbate vascular injury and maladaptive cardiac remodeling over time [47]. Cardiovascular complications, including coronary heart disease (CHD), peripheral arterial disease, and HF, represent the leading cause of morbidity and mortality in individuals with T2DM. Several clinical studies have identified elevated cf-mtDNA as a potential biomarker in T2DM patients with CVD complications, particularly in T2DM patients with CHD [146,147,148,149]. Furthermore, cf-mtDNA correlates positively with C-reactive protein (CRP) level [149] and has been linked to enhanced macrophage activation with interleukin-1 beta and IL-18 secretion, indicating a mechanistic connection to sterile inflammation [148]. In another study, a total of 50 T2DM patients with CHD and 50 healthy participants were recruited. cf-mtDNA concentration was significantly correlated with fasting blood glucose in T2DM patients with CHD, suggesting metabolic stress as a key driver of cfDNA release. Although endothelial injury has been proposed as the primary source of mtDNA in these patients, further study is required to validate this hypothesis. Circulating cfRNAs, such as miRNAs, have also been proposed as prognostic biomarkers in diabetes [150]. A meta-analysis reported that 40 miRNAs are significantly dysregulated in T2DM, with miR-29a, miR-34a, miR-375, miR-103, miR-107, miR-132, miR-142-3p and miR-144 emerging as promising biomarkers for T2DM. The biomarker properties of circulating miRNAs have been further explored in cardiac disease. Their relevance extends to cardiac complications: the study led by Camila Copier et al. [151] identified parallel changes in myocardial and plasma miRNA expression in diabetic mice, and proposed circulating miR-19b and miR-181 as potential biomarkers for diabetic cardiomyopathy progression. Increased levels of lncRNAs, including NKILA [152,153], MALAT1, and MIAT, have also been detected in patients with T2DM [154,155]. Aberrant expression of circulating cirRNAs (e.g., circ_DICAR) has been observed in some CVDs like diabetic cardiomyopathy [156]. Together, these cfDNA and cfRNA signatures show promise as early biomarkers of subclinical diabetic cardiomyopathy. However, current research is limited by small cross-sectional cohorts, heterogeneity in disease severity, and confounding effects of comorbid metabolic syndromes. Large, prospective longitudinal studies integrating cfNA profiling with glycemic control, cardiac imaging, and functional outcomes are needed to define the diagnostic, prognostic, and therapeutic relevance of cfNAs in diabetic cardiomyopathy.

### 5.3. Hypertension

Hypertension, a major risk factor for HF, contributes to both HF with preserved and reduced ejection fraction through chronic pressure overload. This leads to concentric hypertrophy, impaired myocardial relaxation, and progressive myocardial stiffening. Elevated levels of cfDNA are detected in hypertensive patients and are linked to vascular apoptosis and inflammation, reflecting early endothelial injury [157]. These cfDNA profiles may detect vascular dysfunction before echocardiographic abnormalities manifest [157]. Consistent findings are reported in hypertensive disorders of pregnancy, where total cfDNA is markedly increased and correlates with pro-hypertensive factors and adverse clinical outcomes, further supporting cfDNA as a marker of vascular stress and endothelial damage [158]. Circulating cfRNAs have also been found to contribute to the regulation of hypertensive remodeling. MiR-126, a key regulator of endothelial repair and angiogenesis, is reduced in hypertension and associated with impaired vascular regeneration [159]. Additionally, miR-146a has been implicated in the modulation of vascular inflammation, and its elevation may reflect increased atherosclerotic or hypertensive burden [159]. These cfDNA and RNA profiles have led to better mechanistic insights into the maladaptive remodeling process that is associated with chronic hypertension and may be used as diagnostic and prognostic markers in a clinical setting. However, current evidence remains largely associative, and the relative contributions of vascular versus cardiac sources of cfNAs are not well defined. Future efforts should combine tissue-specific cfNA profiling and longitudinal follow-up to establish whether cfNAs can serve as early predictors of hypertensive remodeling and HF transition.

### 5.4. Fibrosis and Adverse Remodeling

Cardiac fibrosis, defined by excessive deposition of ECM components such as collagen I and III, is a central driver of adverse remodeling across diverse HF etiologies. As previously discussed, elevated cfDNA has been linked to myocardial injury and immune activation, both of which amplify profibrotic signaling and promote structural deterioration of the ventricle [140,157]. Circulating cfRNAs, including specific miRNAs, lncRNAs, and circRNAs, have emerged as informative indicators of cardiac fibrosis and HF severity [160]. Multiple circulating pro-fibrotic miRNAs [140,161], such as miR-1 [161,162,163], miR-133 [163], miR-21 [103], miR-208 [164], miR-328 [165], and miR-182 [166], are elevated in association with fibroblast activation, TGF-β/Smad signaling, endothelial-to-mesenchymal transition, and extracellular matrix (ECM) expansion. In contrast, several anti-fibrotic microRNAs, such as miR-29, are suppressed after cardiac injury. For example, miR-29 [167] has been shown to play an antifibrotic role by suppressing genes associated with ECM deposition, and its downregulation has been linked to pathological matrix accumulation in HF [168]. Beyond miRNAs, circulating lncRNAs such as LIPCAR (long intergenic noncoding RNA predicting cardiac remodeling) [169,170], and NRON [171] correlate strongly with left ventricular dilation, myocardial stiffness, and adverse HF outcomes. Circulating circRNAs [30,172] (e.g., circ_0075269, circ_0000284, and circ_0030042 [173]) have been implicated in maladaptive remodeling, particularly in chronic coronary syndrome. These findings suggest that circulating cfRNAs are not only passive biomarkers but also active participants in fibrotic signaling. Longitudinal monitoring of these molecules may enable dynamic monitoring of fibrosis severity, track HF progression, and ultimately guide risk stratification and therapeutic decision-making.

### 5.5. Arrhythmias

Arrhythmias, including atrial fibrillation and ventricular tachyarrhythmias, are common complications in HF patients and significantly increase the risk of sudden cardiac death. Structural remodeling, fibrosis, and ion channel dysregulation all contribute to the development of arrhythmogenesis substrates. Elevated cfDNA levels in patients with arrhythmias may reflect underlying myocardial injury and remodeling [157]. As the most prevalent cardiac arrhythmia, atrial fibrillation (AF) is strongly associated with cardiac dysfunction and thromboembolic events, leading to increased morbidity and mortality. Accumulating evidence indicates that circulating cf-mtDNA is linked to AF onset and progression [80,174,175,176], and may promote arrhythmogenesis by amplifying inflammation, oxidative stress, and electrophysiological instability in the injured myocardium.

In one study, Marit Wiersma et al. quantified cf-mtDNA levels in a cohort comprising 84 controls, 59 cardiac surgery patients in sinus rhythm (SR), 100 paroxysmal AF (PAF), 116 persistent AF (PeAF), and 20 longstanding-persistent AF (LS-PeAF) patients undergoing either cardiac surgery or rhythm control procedures (electrical cardioversion or pulmonary vein isolation) [174]. cf-mtDNA concentration was markedly elevated in PAF patients undergoing AF treatment, with the highest concentrations observed in male patients and in those who later experienced AF recurrence. In contrast, mtDNA levels progressively declined in patients with PeAF and LS-PeAF, consistent with disease stage-dependent changes. An in vitro study further supported that atrial cardiomyocytes contribute to circulating mtDNA, as the cultured tachypaced cardiomyocytes released mtDNA into the culture medium. Similar findings were reported in a separate cohort of 58 AF patients and 10 controls, where elevated cf-mtDNA predicted AF recurrence after catheter ablation [177]. Most recently, Lisa Pool et al. [176] demonstrated that cf-mtDNA levels can help discriminate short- from long-term AF, with lower levels observed in patients with AF duration >3 years. Although this study included patients with diverse cardiac comorbidities, which may influence mtDNA release, the findings further support cf-mtDNA as a potential biomarker for AF staging and recurrence risk.

### 5.6. Atherosclerosis and Vascular Disease

Atherosclerosis, the leading cause of coronary artery disease, contributes to ischemic HF through impaired coronary perfusion, plaque rupture, and microvascular dysfunction. During plaque progression and vascular injury, cfDNA released from apoptotic endothelial cells and activated macrophages contributes to systemic inflammation and may serve as a marker of plaque instability [157]. Circulating miRNAs are increasingly recognized not only as biomarkers of atherosclerotic disease but also as active regulators of vascular pathology. Specific genetic variants in cfNAs, such as the miR-146a rs2910164 CC genotype, have been linked to greater plaque vulnerability in patients with T2DM, especially among older females, those with long-standing diabetes, and those with hypertension [178]. Other dysregulated microRNAs also participate directly in the pathogenic processes of atherosclerosis: miR-210 is elevated both in serum and within atherosclerotic plaques of patients with peripheral artery disease, linking it to hypoxia-related signaling and inflammation [179,180], while miR-223 expression is similarly increased in coronary atherosclerosis and correlates with disease severity [181]. Some cfNAs exhibit context-dependent protective effects, such as miR-155, which is elevated in plasma and atherosclerotic plaques and acts through the miR-155–CARHSP1–TNF-α pathway to dampen chronic inflammation and limit the formation of foam cells, thus reducing plaque formation over time [182]. In contrast, miR-135a-5p and miR-424-5p are downregulated in coronary atherosclerosis, with experimental evidence indicating that their restoration could be protective by inhibiting vascular smooth-muscle cell proliferation and blocking inflammatory mechanisms [183,184]. Given the diverse phenotypes of atherosclerosis, it is likely that distinct cfNA signatures correspond to different stages and anatomical sites of plaque development. Future studies incorporating multi-vascular sampling and longitudinal profiling will be crucial for defining these relationships. Together, these findings position cfNAs as both mechanistic drivers of vascular pathology and promising minimally invasive biomarkers for detecting and stratifying atherosclerotic disease.

Collectively, cfNAs provide valuable mechanistic insight into the pathophysiology of CVDs. Their dynamic release from stressed cardiomyocytes, activated fibroblasts, endothelial cells, and infiltrating immune cells provides a dynamic, systems-level snapshot of the microenvironment in the CVD. Because cfNAs track ongoing cardiac injury and disease progression more accurately than many conventional biomarkers, integrating cfNA signatures with established protein markers and imaging modalities holds strong potential to improve diagnostic precision, prognostic assessment, and personalized management in HF and related conditions.

## 6. Therapeutic Opportunities Targeting cfNAs and Sensing

CfNAs are not merely passive biomarkers but also active mediators of disease progression. Persistently elevated cfNAs act as potent DAMPs, engaging PRRs to sustain inflammation, promote cardiac injury, and drive maladaptive tissue remodeling. Current pharmacologic therapies used for HF primarily focus on hemodynamic stabilization and neurohormonal modulation, functioning as symptom-targeting therapies rather than mechanism-based interventions. These include angiotensin receptor-neprilysin inhibitor/angiotensin-converting enzyme inhibitor/angiotensin receptor blocker (ARNi/ACEi/ARB), beta-blockers, mineralocorticoid receptor antagonists (MRAs), and sodium-glucose cotransporter-2 (SGLT2) inhibitors [185]. Although these therapies alleviate symptoms (e.g., blood pressure, volume overload, sympathetic drive), they do not directly address upstream molecular drivers of injury and inflammation that are increasingly recognized as central to HF progression. To bridge this therapeutic gap, various studies into therapies targeting cfNAs are being widely investigated. These novel mechanism-focused strategies aim to (1) scavenge the circulating cfNAs to reduce their proinflammatory burden; (2) inhibit cfNA release from damaged or stressed cells, and (3) block cfNA-PRR signaling pathways to prevent downstream immune activation.

### 6.1. Enzyme Degradation System

Under physiological conditions, cfNAs are rapidly cleared by plasma nucleases (DNase and RNase) and phagocytic clearance pathways. However, in HF, this balance is disrupted. Excessive release of cfNAs from stressed or dying cells, or impaired clearance mechanisms, leads to their aberrant accumulation and sustained inflammatory signaling. Enhancing cfNA clearance through enzymatic degradation by recombinant DNase and RNase has emerged as a mechanistically targeted therapeutic strategy. Recombinant DNase therapy, such as dornase alfa (Pulmozyme), originally FDA-approved for cystic fibrosis, has shown efficacy in cardiovascular contexts relevant to HF progression. For example, DNase treatment reduced coronary thrombosis and improved microvascular perfusion in MI models, a process directly relevant to post-MI remodeling and HF progression [186,187]. Ge et al. further demonstrated that recombinant DNase I treatment reduced the infarct size, NET formation, MPO activation, and neutrophil accumulation in the heart in ischemia–reperfusion (I/R) model [186]. Similarly, recombinant RNase I treatment has been shown to significantly attenuate myocardial injury and suppress cytokine release in I/R mouse models, suggesting its potential to mitigate cfRNA-driven inflammation in acute decompensated HF [91]. Moreover, in the cardiopulmonary bypass (CPB) model, DNase I treatment significantly reduced plasma cfDNA/NETs levels and ameliorated endothelial dysfunction and inflammation, the key complications that contribute to adverse ventricular remodeling and HF risk [188]. In addition to enzymes, phagocytic cells such as macrophages and dendritic cells also play critical roles in cfNA clearance. Scavenger receptors (e.g., SR-A), complement factors such as C1q, and TAM receptors mediate the uptake of nucleic acid–protein complexes and NET remnants [189,190,191]. Opsonizing molecules, including apolipoproteins ApoE, also promote cfDNA clearance by binding NETs and suppressing inflammation [192,193].

While these innovations mark a significant step toward mechanism-based therapies, DNase therapy also faces significant challenges, such as rapid inhibition by actin binding [194], a short circulating half-life [195], and poor access to nucleosome-protected cfDNA [196]. To overcome these issues, engineered approaches are being developed to enhance enzymatic stability, tissue targeting, and catalytic efficiency in vivo. These therapeutic strategies can be categorized into three complementary approaches: (1) engineering natural nucleases for improved pharmacokinetics and resistance to inhibition; (2) developing synthetic nucleic acid-degrading systems such as nanozymes; and (3) improving delivery methods for targeted tissue penetration. Modified natural nucleases, such as DNase and RNase family members, retain their catalytic specificity while being engineered for improved stability and activity in vivo. For example, PEGylation of DNase has been created to extend its circulation time [197], while actin-resistant DNase variants overcome rapid inhibition by plasma actin [194]. In parallel, synthetic nucleic acid-degrading systems, such as transition-metal-based and polymeric nanozymes [198] display enhanced stability and are more resistant to actin inhibition compared to natural DNases [199]. For example, cerium-based nanozymes have been reported to improve ischemic stroke symptoms and suppress neuroinflammation in murine models by modulating the cGAS-STING axis [198]. Similarly, polymeric DNase-like nanozymes were shown to successfully degrade NETs and suppress neutrophil recruitment in inflammatory bowel disease animal models. Despite these advances, nanozymes generally show reduced catalytic efficiency compared to natural nucleases, and further optimization is required to ensure consistency, safety, and therapeutic efficacy for clinical use.

### 6.2. Binding and Neutralization Strategies

In addition to degrading cfNAs directly, another promising approach involves binding and neutralizing cfNAs before they interact with PRRs. CfNAs are negatively charged due to the phosphate backbone. This makes them prone to being electrostatically captured by positively charged materials such as cationic polymers and nanoparticles. This binding system has demonstrated efficacy in preclinical settings. For instance, cationic nanoparticles neutralize the negatively charged cfNAs and block the TLR9 receptor, thereby attenuating inflammation in acute arthritis animal models [200]. Although cf-NA-binding systems have not yet been widely evaluated in HF, their mechanistic relevance is applicable. In HF, cf-mtDNA engages TLR9 in cardiomyocytes and macrophages, thereby amplifying inflammation and cardiac remodeling [201]. However, these approaches also face persistent limitations. One challenge is that the strong positive charge of nanoparticles will also allow them to interact nonspecifically with serum proteins or cell membranes, leading to cytotoxicity and off-target effects [202]. Excessive positive charge is detrimental to cell membrane stabilization, which can trigger complement activation and further exacerbate inflammation. Another major limitation is the delivery challenge. These nanoparticles are often rapidly cleared by the mononuclear phagocyte system and tend to accumulate in the liver and spleen, limiting their bioavailability in cardiovascular tissues [14]. Future work should optimize the dosing strategy and long-term safety of DNase therapy and extend current findings to models with longitudinal follow-up to determine whether nuclease-based cfNA clearance provides durable benefits.

### 6.3. Advanced Delivery Approaches

Effective cfNA-targeted interventions require optimized delivery methods to improve tissue targeting and minimize systemic toxicity. Stimuli-responsive systems, such as pH or ROS-sensitive linkers, enable the controlled release of therapeutic agents precisely and activate therapy specifically within inflammatory or ischemic tissues. However, because pH or ROS vary widely in disease microenvironments, these systems face limitations in achieving consistent and predictable drug release [203,204]. Most recently, exosome-based systems have emerged as a promising biologically compatible delivery method [205,206]. Unlike synthetic carriers, exosomes are naturally derived vesicles that contain a native lipid bilayer and surface proteins. This biological similarity confers several advantages: it protects the therapeutic cargo from degradation by the phagocyte system, prevents complement activation, and reduces the risk of being targeted by neutralizing antibodies. Nordmeier et al. demonstrated that exosomes isolated from a cancer cell line and loaded with nanoparticles were able to evade immune detection and nuclease degradation while maintaining high delivery efficiency [207]. Another group engineered fusion protein exosomes containing a specific RNA-binding protein and an exosome membrane protein, which successfully recruited specific RNA sequences for degradation, suppressing inflammatory gene expression, and preventing liver fibrosis [208]. These findings suggest that exosomes represent a more physiologically adaptable and translationally feasible delivery platform. By enhancing both specificity and stability, exosome-based systems may facilitate targeted delivery of cfNA-modulating therapies to sites of cardiovascular injury. However, it also presents several limitations. First, current loading techniques remain technically challenging and show considerable variability [207], and natural exosomes inherently carry endogenous RNAs that may cause off-target effects [208]. In addition, exosome uptake differs markedly across cell types, which may limit delivery efficiency in peripheral cardiovascular tissues [207].

### 6.4. Nucleic Acid-Targeted Gene Silencing Therapies

Gene silencing approaches such as antisense oligonucleotides (ASOs) and RNA interference (RNAi) provide targeted approaches to inhibit cfNA sensing and downstream inflammatory signaling. Valentin et al. designed sequence-dependent 2′-O-methyl (2′OMe) gapmer ASOs to target DNA sensors such as cGAS and TLR9, resulting in reduced inflammatory cytokine production and tissue injury [209]. Given that activation of cGAS–STING and TLR9 pathways by cfDNA contributes to maladaptive remodeling and adverse outcomes after MI, these ASO-based strategies represent a mechanistically targeted approach to mitigating inflammation-driven progression to HF. In another study, Liu et al. generated a nano-bioconjugate combining antibodies and ASOs to suppress mTOR expression in macrophages, thereby promoting autophagy and alleviating atherosclerotic disease pathology [210]. Although ASOs have not been widely applied to target cf-NA sensors in cardiovascular diseases specifically, their modular design, sequence-specific targeting, and low off-target potential underscore their strong therapeutic promise for future cardiovascular applications.

RNAi-based strategies such as small interfering RNAs (siRNAs) have also demonstrated efficacy in modulating cfNA-related pathways. Ye et al. employed siRNA-mediated silencing of Caveolin-1 (CAV-1) in mouse neutrophils, which was proven to successfully suppress NET formation and decrease ROS levels [211]. Inhibition of P2Y6R, a receptor involved in neutrophil recruitment and NET formation, attenuated inflammatory cell migration via the IL-8/CXCR2 pathway [212]. Moreover, Liu et al. further developed a PEGylated cationic nanoparticle delivering siRNA against RAGE (a receptor involved in NET formation), and observed improved myocardial recovery in I/R models by inhibiting inflammatory mediators [213]. Most recently, nanocomplexes coated with neutrophil membrane carrying siRNA against integrin α9 significantly reduced infarct size, neutrophil infiltration, and NET formation in MI models [214]. Although siRNA can modulate NET formation by targeting signaling pathways, these regulatory mechanisms are largely disease specific. Future studies should be performed to delineate additional NET-activating pathways relevant to cardiovascular conditions and to clarify the functional roles of NET-derived cfNAs in cardiac pathology. Together, these findings highlight the potential of gene silencing therapies to selectively disrupt cfNA sensing, and NET-associated inflammation. By targeting upstream molecular drivers of cardiac injury, ASOs and siRNAs represent a promising class of mechanism-based interventions for HF and related CVDs.

## 7. Emerging Frontiers: Microbial cfDNA and Gut–Heart Axis

While most cfNA studies in CVD have focused on host cell-derived cfNAs, emerging evidence has identified a subset of cfDNA, commonly referred to as circulating microbial cfDNA or bacterial DNA [215,216]. Detected through high-sensitivity sequencing approaches such as 16S rRNA sequencing, microbial cfDNA fragments have been found in significantly higher levels in CVD patients compared to healthy controls [215,217]. These microbial-derived DNAs likely originate from bacterial translocation across the compromised mucosal or endothelial barriers, such as the leaky gut, and enter the circulation, where they act as potent PAMPs. Upon engagement with PRRs, microbial cfDNA amplifies inflammation and endothelial injury, the key processes driving atherosclerosis, cardiac remodeling, and HF progression. Moreover, distinct quantitative and compositional microbial cfDNA profiles have been observed among patients with acute coronary syndrome, stable coronary disease, and healthy individuals [218]. Elevated microbial cfDNA levels correlate with systemic inflammatory mediators such as IL-6, C-reactive protein (CRP), lipopolysaccharide (LPS), and altered short-chain fatty acid (SCFA) metabolism, linking gut dysbiosis to cardiovascular risk [215]. Mechanistic studies support a direct pathogenic role: aging mouse cardiomyocytes have accumulated gut microbial DNA, triggering inflammatory signaling and contractile dysfunction. Moreover, exposure of young cardiomyocytes to this microbial DNA recapitulated these inflammatory responses and contractility defects, highlighting its pathogenic impact. However, blocking microbial DNA infiltration attenuated these pathogenic effects of microbial DNA on cardiac function [219]. The effects of the gut–heart axis in cardiac remodeling are further supported by multiple animal studies, including rat hypertension models [220], rat cardiac hypertrophy model [221], rat heart failure models [222], rat myocardial infarction model [223], and mouse cardiac hypertrophy model [224]. Notably, gut health interventions, including dietary modifications, prebiotics, and probiotics, show clinical benefit in CVD patients [225]. Collectively, these findings establish microbial DNA leakage as both a biomarker of barrier dysfunction and a mechanistic driver of cardiovascular injury, expanding the conceptual landscape of cfNA biology. This emerging area also highlights the need to address key mechanistic and translational challenges, such as distinguishing host vs. microbial cfNA sources, standardizing detection methods, and validating clinical relevance.

## 8. Limitations and Challenges

Despite promising advances in circulating cfRNA and cfDNA profiling for cardiovascular disease, several important limitations remain.

### 8.1. Clinical Cohort Design and Clinical Confounders

Most available studies are based on small, retrospective, or case-enriched cohorts with limited representation of real-world clinical populations. Future work should include well-powered cohorts that reflect the full range of patients with CVDs, including those with potential alternative diagnoses. This is particularly important given that major comorbidities such as chronic kidney disease, systemic inflammatory states, active malignancy, pregnancy, and recent trauma can independently elevate cfNAs and act as significant confounders. Incorporating rigorous adjustment for these variables will be critical to establish the true incremental diagnostic and prognostic value of cfNAs.

### 8.2. Biological Interpretation

Biological interpretation of cfNAs remains challenging. Analytical variability across platforms, heterogeneous cellular origins, and limited validation in large and diverse patient cohorts all contribute to inconsistent findings. Moreover, the causal and mechanistic roles of cfNAs in HF are still not fully defined. These challenges are compounded by the incomplete reference atlases capturing cfNA signatures from key cardiac cells, such as cardiomyocytes, fibroblasts, endothelial cells, and immune cells, which limit the precise interpretation of circulating cfNAs. Although cardiomyocyte-enriched cfNAs have emerged as promising candidate biomarkers, biological specificity remains a major limitation. Many non-cardiac conditions can result in substantial release of nucleic acids into the circulation, and the proinflammatory properties of free RNAs and mitochondrial DNA are not unique to myocardial injury. Consequently, cfNAs alone are unlikely to achieve robust diagnostic performance without integration with additional molecular or clinical context. Addressing these gaps will require longitudinal studies integrating cfNA profiling with multimodal clinical data, including imaging, myocardial biopsy, clinical risk scores, and patient outcomes, to clarify temporal dynamics and clinical relevance. Incorporation of molecular features, such as methylation signatures or splice isoform analysis, can also help in distinguishing myocardial injury from systemic tissue damage. Mechanistic evidence should be further strengthened through human cardiac models, such as human iPSC-derived organoid systems.

### 8.3. Clinical Feasibility and Assay Turnaround Time

Clinical feasibility represents another major barrier, particularly in acute care settings. Although sequencing-based and quantitative transcriptomic platforms continue to improve in sensitivity, current cfNA workflows remain too slow for time-critical conditions. In emergency cases such as acute coronary syndrome or acute heart failure, diagnostic biomarkers must be readily available and provide actionable results within minutes. Existing cfRNA and cfDNA assays, which require plasma separation, extraction, and multi-step amplification or sequencing, cannot match the turnaround time of established clinical biomarkers such as cTns or natriuretic peptides. If robust cell- and tissue-specific cfDNA and cfRNA reference maps can be established, it may become feasible to transition from sequencing-based assays to targeted, rapid detection platforms. In principle, microfluidic or chip-based systems with sequence-specific probe arrays [226,227] or CRISPR-based sensors [228,229,230] could function analogously to an “ELISA for nucleic acids,” enabling multiplexed, tissue-of-origin-specific cfDNA/cfRNA detection in just a few minutes. These methods could substantially shorten detection time and bring cfNA-based diagnostics methods closer to the turnaround times of traditional protein markers.

## 9. Conclusions

cfNAs represent a distinct class of mechanism-linked biomarkers that provide real-time, noninvasive insight into cardiac injury, inflammation, and tissue remodeling. By capturing upstream molecular events, from early cardiac cell stress to immune activation, cfNAs hold strong potential for earlier diagnosis, improved risk stratification, and therapeutic monitoring across CVDs. Ongoing advances in scalable and user-friendly detection technologies, together with standardized pre-analytical workflows, will facilitate broader clinical application. Continued progress in methodological standardization, advanced analytical platforms, and interdisciplinary clinical research is steadily addressing current limitations. With further technological refinement and rigorous clinical validation, cfNAs have the potential to significantly improve diagnostic accuracy, enable personalized risk stratification, and support the development of targeted, mechanism-based therapies for HF and related CVDs.

## Figures and Tables

**Table 1:** Information on intracellular nuclear DNA and cell-free DNA (nuclear origin).

Feature	Cellular Nuclear DNA	Circulating cf-DNA (Nuclear Origin)
Location	Nucleus	Extracellular compartment, circulating in plasma and other biofluid [22]
Size	40–240 million bp per chromosome	35–1000 bp, typically ~166 bp (mono-nucleosomal fragments) [31,32]
Packing	Condensed into chromatin, wrapped by histones	Protected by histones (nucleosomes) or extracellular vesicles [33]
Stability	Highly stable in nucleus; slow degradation	Rapid turnover (half-life 4 min–2 h), stability enhanced by protein/vesicle binding [34]
Release Mechanisms	Retained intracellular under normal conditions	Released via apoptosis, necrosis, active secretion, Extracellular traps (NETosis), or microbe [35]
Isolation Techniques	Standard DNA extraction/precipitation	Column-based isolation and purification from biofluids [36]
Clinical Application	Genomics, genetic testing	Noninvasive diagnostics (prenatal testing, oncology, transplant monitoring, cardiovascular disease) [37,38]

**Table 2:** Information on intracellular mitochondrial DNA and cell-free DNA (mitochondrial origin).

Feature	Cellular Mitochondrial DNA	Circulating cf-mtDNA (Mitochondrial Origin)
Location	Mitochondria	Extracellular compartment, circulating in plasma and other biofluids [39]
Size	Circular, 16.6 kb genome	Short fragment (<1 kb), highly variable [39,40]
Packing	Naked, histone-free	Histone-free; may be packaged in vesicles or bound to proteins [40]
Stability	Moderately stable in mitochondria	Unstable in circulation due to lack of histone protection [39]
Release Mechanisms	Release during mitochondrial stress or cell death	Released via apoptosis, necrosis, active secretion, Extracellular trap (NETosis) [35]
Isolation Techniques	Mitochondria DNA isolation kits	Column-based EV-associated extraction from plasma [36]
Clinical Application	Mitochondrial genetics, inherited disease testing	Biomarker for oxidative stress, inflammation, adverse outcomes in cardiovascular diseases [41]

**Table 3:** Information on intracellular RNA and cell-free RNA (nuclear/cytoplasmic origin).

Feature	Cellular Nuclear RNA (Nuclear/Cytoplasmic Origin)	Cellular cfRNA (Nuclear/Cytoplasmic Origin)
Location	Nucleus & Cytoplasm	Extracellular compartment, circulating in plasma and other biofluids [42]
Size	miRNA ~18–24 nt; mRNAs & IncRNAs up to >100 bp	Mostly 20–200 bp (can up to ~500 bp), fragments [43]
Packing	RNA-binding proteins, ribosomes, secondary structures	Encapsulated in Evs (exosomes, microvesicles), bound to proteins or lipoproteins [44]
Stability	Relatively unstable (degraded by Rnases without protection)	Short half-life; stability improved when vesicle- or protein- protected [45]
Release Mechanisms	Synthesized & retained intracellularly	Released via apoptosis, necrosis, active secretion, EV transport [46]
Isolation Techniques	Standard RNA extraction	Circulating and exosomal RNA isolation kits [36,43]
Clinical Application	Tumor biology, drug resistance, gene expression studies	Noninvasive biomarkers for cardiovascular disease, fibrosis, inflammation [47]

**Table 4:** Information on intracellular RNA and cell-free RNA (mitochondrial origin).

Feature	Cellular Mitochondrial RNA	Cellular cf-mtRNA (Mitochondrial Origin)
Location	Mitochondria (transcribed from mtDNA)	Extracellular compartment, circulating in plasma and other biofluids
Size	mRNAs, tRNAs, rRNAs (hundreds of thousands of nt)	Typically short fragments (<400 nt), unstable unless vesicle/protein bound [43]
Packing	Associated with mitochondrial ribosomes	Detected in extracellular vesicles or protein complexes [46]
Stability	Relatively stable in mitochondria	Highly unstable; quickly degraded unless vesicle-protected [48]
Release Mechanisms	Released during mitochondrial stress, apoptosis	Released under stress, apoptosis, necrosis [49]
Isolation Techniques	Standard RNA extraction	Circulating and exosomal RNA isolation kits [50,51]
Clinical Application	Study of mitochondrial function, oxidative stress	Biomarkers for CVD, Alzheimer’s disease, preeclampsia, renal disease [52,53,54,55]

## Data Availability

No new data were created or analyzed in this study.
